# Effectiveness of Milieu Therapy in reducing conflicts and containment rates among schizophrenia patients

**DOI:** 10.17533/udea.iee.v38n1e06

**Published:** 2020-02-26

**Authors:** Sandhya Bhat, Sreevani Rentala, Raveesh Bevinahalli Nanjegowda, Xavier Belsiyal Chellappan

**Affiliations:** 1 Staff Nurse, District Hospital, Dharwad, Karnataka, India. Email: sandhyabhat1973@gmail.com District Hospital India sandhyabhat1973@gmail.com; 2 Nursing Professor and Head, Dharwad Institute of Mental health and Neuroscience, Karnataka, India. Email: sreevani.phd@gmail.com. Corresponding author Dharwad Institute of Mental health and Neuroscience Karnataka India sreevani.phd@gmail.com.; 3 Professor and Head, Dept. of Psychiatry, Mysore Medical College, Mysore, India. Email: raveesh6@yahoo.com Mysore Medical College Mysore India raveesh6@yahoo.com; 4 Assistant Professor, College of Nursing, AIIMS Rishikesh, India. Email: jinbelsi@gmail.com College of Nursing, AIIMS Rishikesh India jinbelsi@gmail.com

**Keywords:** schizophrenia, inpatients, psychiatric department, hospital, milieu therapy, aggression, self-injurious behavior, esquizofrenia, pacientes internos, servicio de psiquiatría en hospital, terapia ambiental, conducta autodestructiva., esquizofrenia, pacientes internados, unidade hospitalar de psiquiatría, terapia ambiental, comportamento autodestrutivo.

## Abstract

**Objective.:**

To evaluate effectiveness of Milieu Therapy in reduction of conflict and containment rates among schizophrenia patients.

**Methods.:**

This study utilized quasi experimental non-equivalent control group pre-post design. One hundred schizophrenia patients admitted in acute psychiatric wards were non-randomly assigned to either of the experimental (*n*=50) or control group (*n*=50). The experimental group received both milieu therapy and routine hospital treatment. The Milieu Therapy intervention Included environmental modification and structuring ward activities, establishing effective interaction with patient, and teaching caregivers on managing conflict behavior of patient. The control group received only routine treatment in the hospital. Outcome measures on conflict and containment rates were evaluated for both the groups at baseline and at 2^nd^, 3^rd^ and 15^th^ day. The Patient-Staff Conflict Checklist Shift Report (PCC-SR) was used to collect information about rates of conflict and containment.

**Results.**

Compared with control group, the experimental group participants showed decrease in aggressive behavior, self-harm behavior and general rule breaking behavior at baseline and 2^nd^, 3^rd^ and 15^th^ day (F=4.61, *p*<0.004, *η* ^2^=0.04; F=11.92, *p*<0.001, *η* ^2^=0.11; F=6.94, *p*<0.001, *η* ^2^=0.06) over seven days interval.

**Conclusion.:**

The present study findings provided evidence for the effectiveness of integrating Milieu Therapy in psychiatric acute wards in reducing conflict behaviors among schizophrenia patients. Milieu therapy should be considered as an integral part of psychiatric care settings in these patients.

## Resumen

**Objetivo.:**

Evaluar la efectividad de la terapia Milieu en la reducción de las tasas de conflicto y contención entre los pacientes con esquizofrenia.

**Métodos.:**

Este estudio utilizó un diseño cuasi experimental con grupo control no equivalente y evaluación pre y posintervención. Cien pacientes con esquizofrenia hospitalizados en salas psiquiátricas en un hospital público en Karnataka (India) se asignaron de forma no aleatoria a los grupos experimental (*n*=50) y control (*n*=50). El grupo experimental recibió la terapia de Milieu, además del tratamiento hospitalario de rutina. La terapia de Milieu incluyó la modificación ambiental y la estructuración de las actividades de la sala, el establecimiento de una interacción efectiva con el paciente y la enseñanza a los cuidadores sobre el manejo del comportamiento conflictivo del paciente. El grupo control recibió solamente el tratamiento de rutina en el hospital. Las medidas de resultado (tasas de conflictos y de contención) se evaluaron en ambos grupos en los días de inicio, 2º, 3º y 15º posadmisión. El *Patient-Staff Conflict Checklist Shift Report (PCC-SR)* se utilizó para la recolección de la información acerca de las tasas de conflicto y contención.

**Resultados.:**

En comparación con el grupo de control, los participantes del grupo experimental mostraron una disminución en el comportamiento agresivo, el comportamiento de autolesión y el comportamiento de incumplimiento de las reglas generales al inicio y al segundo, tercer y décimo quinto día (F=4.61, *p*<0.004, *η*2=0.04; F=11.92, *p*<0.001, *η*2=0.11; F=6.94, *p*<0.001, *η*2=0.06).

**Conclusión.:**

Los hallazgos del presente estudio proporcionaron evidencia de la efectividad de la Terapia Melieu en la reducción de comportamientos conflictivos en el paciente esquizofrénico hospitalizado en fase aguda. La Terapia Milieu debe considerarse como una parte integral en los entornos de atención de estos pacientes.

## Resumo

**Objetivo.:**

Avaliar a eficácia da terapia Milieu na redução das taxas de conflito e contenção entre pacientes com esquizofrenia.

**Métodos.:**

Este estudo utilizou um desenho quase-experimental com um grupo controle não equivalente e avaliação pré e pós-intervenção. Cem pacientes com esquizofrenia hospitalizados em salas psiquiátricas em um hospital público de Karnataka (Índia) foram designados não aleatoriamente para os grupos experimental (n = 50) e controle (n = 50). O grupo experimental recebeu terapia Milieu, além de tratamento hospitalar de rotina. A terapia de Milieu incluiu modificação ambiental e estruturação das atividades da enfermaria, estabelecendo uma interação efetiva com o paciente e ensinando os cuidadores sobre o gerenciamento do comportamento conflitante do paciente. O grupo controle recebeu apenas tratamento hospitalar de rotina. As medidas de resultado (taxas de conflito e contenção) foram avaliadas em ambos os grupos nos dias de início, 2, 3 e 15 após a admissão. O Relatório de Mudança de Lista de Verificação de Conflitos entre Pacientes e Funcionários (PCC-SR) foi usado para coletar informações sobre taxas de conflitos e contenção.

**Resultados.:**

Comparados ao grupo controle, os participantes do grupo experimental mostraram uma diminuição no comportamento agressivo, no comportamento autolesivo e no não cumprimento das regras gerais no início e no segundo, terceiro e décimo quinto dia (F = 4,61, p <0,004, η2 = 0,04; F = 11,92, p <0,001, η2 = 0,11; F = 6,94, p <0,001, η2 = 0,06).

**Conclusão.:**

Os achados do presente estudo forneceram evidências da eficácia da Terapia Melieu na redução de comportamentos conflitantes no paciente esquizofrênico hospitalizado na fase aguda. A terapia Milieu deve ser considerada como parte integrante do ambiente de atendimento desses pacientes.

## Introduction

Conflict and containment rates are associated with a primary diagnosis of schizophrenia.(1) Conflict refers to any patient action that threatens patient or staff safety which may include physical violence, verbal aggression, go absconding, use of alcohol or illegal substances, self harm and medication refusal. Containment refers to any method that psychiatric staffs use to prevent or manage the conflict event, such as seclusion, special observation, de-escalation, time-out, manual restraint and enforced medication.(2) 

In contrast to other hospital environments, within psychiatric inpatient settings, patient risk is conceptualized as affecting not only the individual, but also other patients, staff and the general public, widening the sphere of risk.(3) Conflicting behavior in acute psychiatric wards can be a major problem, not only because of the potential injury it may cause to the patients and staff, but also because of the counter therapeutic effects of both violent behaviors and strategies to prevent such behavior. Many hospitals are using pharmacological interventions to manage conflicting behaviors.(4) Although, psychosocial therapies have proved to be effective in managing conflicting behaviors, pharmacological interventions are continued to be widely used. These conflict and containment are important matters for hospital management and nursing practice.(5) 

Bowers(6) revealed a set of interventions that can increase safety in psychiatric wards. These interventions reduced aggression, self harm and other risky behaviours by 15% and reduced coercive controls such as restraints by 24%. Creating a therapeutic milieu is a basic intervention in mental health nursing practice, and is inclusive of everything in the immediate inpatient environment. Everything in the milieu is meant to promote healing, and includes the staff, the physical structure of the unit and the emotional climate of the staff and patients on the unit.(7)

A nurse in a psychiatric ward is the responsible person for providing therapeutic environment such as providing the chance for the individuals in expressing feelings, determining the risks of harming self or others, providing a secure and comfortable physical environment.(8) A major challenge in psychiatric inpatient care is to create an environment that promotes patient recovery, patient safety and good working environment for staff. Staff members need to implement safe interventions to patients and gradually bring back responsibility and initiative to the patient.(9) At the same time violence in the ward may negatively affect patient recovery(10,11) staff health,(12,13) and the organization.(14) Therefore, it is important to create a safe environment through primary preventive interventions so that both staff and patient can feel safe. Evidences showed that psychiatric nurses also adopted milieu concepts in inpatient psychiatric settings in western countries^.^(7)

Milieu therapy interventions are simple, safe, cost-effective and can be used in any inpatient psychiatric settings. For implementing milieu therapy nurses, do not require any specialized training. However there is no known literature on effectiveness of implementing milieu therapy in psychiatric wards in Indian context. The present study was conducted with this background, to test the effectiveness of milieu therapy on conflict and containment rates among schizophrenia patients.

## Methods

Study design. This study utilized quasi experimental non-equivalent control group pre-post design.

Setting. The present study was conducted at acute psychiatric wards of state government hospital, Karnataka, India. It is a 375 bedded hospital with 20 bedded 4 acute wards. The main objective of this institution is to provide quality services to patients. The clinical services consist of in-patient, outpatient, emergency and rehabilitative services. The 20-bed inpatient acute wards offer a comprehensive treatment program including pharmacology and psychosocial treatment. The in-patient treatment usually spans for 10 to 15 days.

Sample and sampling technique. A total of 122 patients were admitted during the data collection period, 102 met inclusion criteria, 2 participants declined to participate. A total of 50 participants each were selected for the experimental and control group using convenient sampling technique. To prevent intervention contamination the subjects who admitted in two in-patient wards selected for control group and another two in-patient wards for experimental group.

Ethical considerations. This study was approved by the Institutional Ethical Committee. Participants and care givers were informed about the purpose, time duration of therapy sessions and follow-up assessments. Subsequent to explanation of the benefits and risks of the study the participants/caregivers gave their written consent.

**Diagram 1:**
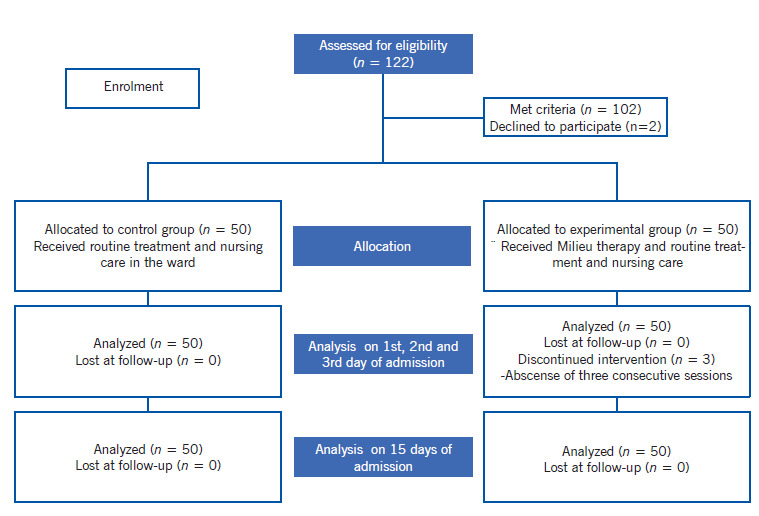
Flow chart

Subject recruitment. Inclusion criteria were age between 20-60 years and a diagnosis of schizophrenia made by a psychiatrist based on ICD 10 criteria with a recommendation for inpatient management. The study excluded patients with co morbid medical disorders, those not willing to stay in hospital for minimum 15 days and those admitted in chronic wards. After obtaining formal permission from the institutional authority recruitment of subjects took place at acute wards. Data was collected between December 2016 and May 2017 in acute psychiatric wards. On an average, 5 to 6 patients were recruited in a week. To prevent intervention contamination, the patients admitted in one male and one female ward was selected for experimental group and those in other wards for control group. Subject allocation to control and experimental group presented in Diagram 1.

Data collection and intervention. Initially patients who diagnosed with schizophrenia and also on antipsychotics were identified. Each patient was contacted and a personal interview was arranged for baseline assessment which included socio-demographic details, clinical characteristics, conflict and containment rates. After the initial assessment, participants in experimental group underwent milieu therapy along with routine treatment and nursing care. The control group participants received the routine treatment and nursing care offered at wards. 

*Routine treatments and nursing care.* It included pharmacological management and routine nursing care. The routine nursing care includes medication management, providing psycho-education on individual basis, care of activities of daily living, involving recreational and diversional activities of the patient, etc.

*Milieu therapy.* Milieu therapy was provided to experimental group participants throughout their admission period. Therapy was provided by first author who was a registered nurse. Intervention was given on an individual basis to participants and as group approach to the caregivers. The first author observed the patients in the ward from morning 8 am to evening 4 pm, a total of 8 hours from day 1 to day 15 and noted conflict and containment rates among patients and implemented therapy. The therapy includes environmental modification, structuring ward activities, effective interaction with patient and teaching caregivers on managing conflict behavior of the patient. The details of these interventions were described in Table 1. 

**Table 1:** Details of Milieu Therapy

Content	Objectives	Activities involved
Environmental modification	To provide safe and secure environment	Removing sharp objects from the patient environment
Structuring ward activities	Provide structured activity schedule	Preparing activity schedule for activities of daily living, physical exercise, breakfast, lunch and dinner, rest, diversional activities such as music, craft and drawing etc.
		Displaying ward rules
Effective interaction with patient	To establish rapport with the patient and the caregivers	Listening to patient
	To provide supportive environment for venting negative emotions	Encouraging patient and caregivers to express their feelings
	To provide positive feedback for adaptive behaviour	Teaching on safe and unsafe behavior
		Recognizing adaptive behavior and provide positive reinforcement
		Encouraging the patient to follow structured schedule for their routines
Teaching caregivers on managing conflict behavior of the patient	Enable the caregivers to manage conflict behaviours of their patients	Explaining on various conflicting behaviors and their triggers and consequences.
		Describing on effective communication to deal with these conflicting behaviors

Content validity of the intervention module was assessed by a panel of 10 subject experts. Panel members were asked to assess the content of the therapy module for its appropriateness and use among schizophrenia patients. A few suggestions were given by them to ensure that it was better tailored to the needs of patients; these were subsequently incorporated into the module. 

Post assessment. Conflicts and containment rates were assessed using patient staff conflict observational check list on 1^st^, 2^nd^, 3^rd^ and 15^th^ days of admission. 

Measures. At treatment entry, information was obtained about participants’ socio-demographics, clinical history, conflict and containment rates. Before administration of questionnaires to study participants, the questionnaires were pretested in a similar setting for suitability and reviewed by experts for accuracy. *1)*Socio demographic information comprised of basic information such as age, gender, marital status, educational status, religion, area of residence, type of family and monthly income. Clinical history comprises of age of onset of illness, duration of psychiatric illness, duration of treatment, number of previous hospitalizations, ECT details, and family history of mental illness. 2) *Conflict and containment rates* were assessed using patient staff-conflict check list (PSCC). It is an observational checklist consisting of 30 items, 21 items related to 6 conflicting behaviors and 9 items related to containment measures. Based on the observations a score was assigned for each of the conflicting behaviors and the containment measures used by the staff nurse to control such behavior. These scores were added to obtain the frequency of that behavior, with higher scores indicating greater frequencies and increased conflicting and containment rates for the patients. The inter-rater reliability demonstrated a satisfactory Kappa of 0.69.(15)

Data analysis. Baseline characteristics of the control and experimental groups were compared using chi-square or independent t-test for categorical or continuous variables respectively. The changes in the outcome variables from baseline to 15-days were compared using repeated measure analysis of variance. Partial eta-square ((^2^) was calculated as the effect size of major statistical tests were based on Cohen’s suggestions. 

## Results

Comparison of baseline socio-demographic variables between groups showed that both the groups were comparable in terms of their baseline, clinical and on outcome variables, except for duration of treatment with antipsychotics and family history of mental illness. Significantly higher number of subjects in control group (*n*=30) had family history of mental illness. There were significantly higher number of subjects in experimental group (*n*=23) with duration of treatment more than 3 years (Tables 2 and 3).

**Table 2:** Baseline comparison of socio-demographic variables between the groups

Variables	Variables	Group	Group	t\χ^2^	***p*-value**
		Control (*n*=50)	Experiment (*n*=50)		
Age	20-29 years	18	21	1.131	0.568
	30-39	19	14		
	40 and above	13	15		
Gender	Male	26	22	0.641	0.423
	Female	24	28		
Marital status	Single	16	17	0.045	0.832
	Married	34	33		
Education	Illiterate	12	16	0.97	0.819
	Primary	16	14		
	Secondary	10	8		
	PUC and above	12	12		
Religion	Hindu	44	45	0.102	0.749
	Muslim	6	5		
Residence	Rural Urban	39 11	44 6	1.772	0.183
Type of family	Nuclear	33	32	0.044	0.834
	Joint	17	18		
Monthly family income	≤Rs.5 000	16	10	2.357	0.308
	Rs.5 001 to 10 000	23	30		
	Rs.10 001and above	11	10		

Note: Rs. 70 =1 US Dollar

**Table 3:** Baseline comparison of clinical characteristics and outcome variables between groups

Clinical characteristics	Clinical characteristics	Group	Group	χ^2^/ *t*	***p*-value**
		Control (*n*=50)	Experiment (*n*=50)		
Age at onset of illness	20-29	26	33	2.140	0.343
	30-39	14	9		
	40 and above	10	8		
Duration of present illness	Up to 3months	31	28	0.626	0.731
	3 to 9 months	8	11		
	More than 9 months	11	11		
Duration of treatment with antipsychotics	Not treated	0	15	30.366	0.0001*
	Up to 3 months	16	2		
	3months to 3years	19	10		
	More than 3years	15	23		
No. of previous hospitalizations	No	26	29	3.355	0.3400
	1time	15	9		
	2times	3	7		
	3 and above	6	5		
ECT details	No	36	40	0.877	0.349
	Yes	14	10		
Family history of mental illness	No	20	35	9.091	0.0030*
	Yes	30	15		
Conflict rates -Mean (SD)	Conflict rates -Mean (SD)	9.84	20.40	-0.27	0.78
Conflict rates -Mean (SD)	Conflict rates -Mean (SD)	(7.90)	(12.11)		
Containment rates -Mean (SD)	Containment rates -Mean (SD)	02.02	02.04	-0.15	0.88
Containment rates -Mean (SD)	Containment rates -Mean (SD)	(0.51)	(0.78)		

The changes in outcome variables from baseline to 15 days between the groups showed that there was a significant milieu therapy interventions effect in aggressive behavior, self-harm and general rule breaking between two groups. Compared with the control group, the experimental group showed statistically significant reduction in aggressive behavior, self-harm and general rule breaking, among patients with schizophrenia *o*ver 15 days of time (Table 4).

**Table 4:** Comparison of conflict and containment rates from baseline to 15^th^ day

Time of assessment	Control group Mean (SD)	Experimental group Mean (SD)	Group x Time F-value	*p-value*	(^2^
Aggressive behavior					
Baseline (T0)	9.18 (4.18)	9.76 (6.92)	4.61	<0.001	0.04
Day 2 (T1)	6.48 (3.62)	6.80 (5.84)			
Day 3 (T2)	4.50 (3.98)	4.14 (4.81)			
Day 15 (T3)	3.06 (2.97)	1.76 (2.92)			
Self-harm					
Baseline (T0)	0.68 (0.84)	1.83 (2.10)	11.92	<0.001	0.11
Day 2 (T1)	0.66 (0.77)	1.29 (1.98)			
Day 3 (T2)	0.40 (0.63)	0.39 (1.16)			
Day 15 (T3)	0.22 (0.41)	0.22 (0.85)			
General rule breaking					
Baseline (T0)	6.98 (3.17)	5.22 (3.13)	6.94	<0.001	0.06
Day 2 (T1)	3.70 (2.23)	3.52 (2.44)			
Day 3 (T2)	3.22 (2.61)	1.52 (1.92)			
Day 15 (T3)	2.18 (2.87)	0.74 (1.44)			
Drugs or alcohol use					
Baseline (T0)	0.50 (0.50)	0.76 (0.77)	3.11	0.27	0.31
Day 2 (T1)	0.46 (0.50)	0.42 (0.49)			
Day 3 (T2)	0.14 (0.35)	0.06 (0.23)			
Day 15 (T3)	0.00 (0.00)	0.04 (0.11)			
Absconding behavior					
Baseline (T0)	1.08 (0.63)	1.10 (0.81)	1.83	0.14	0.01
Day 2 (T1)	0.36 (0.48)	0.52 (0.54)			
Day 3 (T2)	0.24 (0.43)	0.12 (0.32)			
Day 15 (T3)	0.08 (0.27)	0.16 (0.37)			
Medicine related behavior					
Baseline (T0)	1.42 (0.75)	1.60 (0.69)	1.37	0.251	0.01
Day 2 (T1)	1.20 (0.60)	1.00 (0.67)			
Day 3 (T2)	0.94 (0.71)	0.92 (0.92)			
Day 15 (T3)	0.32 (0.58)	0.28 (0.53)			
Containment Measures					
Baseline (T0)	2.02 (0.51)	0.04 (0.78)	1.38	0.24	0.01
Day 2 (T1)	1.50 (0.50)	1.74 (0.82)			
Day 3 (T2)	1.32 (0.51)	1.48 (0.78)			
Day 15 (T3)	0.90 (0.30)	0.88 (0.62)			

## Discussion

Overall, the participants in experimental group showed marked decrease in aggressive behavior, self-harm and general rule breaking behavior. This suggests that milieu therapy interventions added to routine care could be regarded as an additional benefit in the treatment of schizophrenia patients. The findings are in accordance with earlier research which documented that simple interventions aiming to improve staff relationship with patients can reduce the frequency of conflict and containment behaviors among patients.(7)

In the present study, participants in milieu therapy were ensured safe and secure environment, observed constantly and provided structured ward activities to reduce aggression among patients with schizophrenia. Previous study showed that small changes in routine practices of psychiatric ward reduced the conflict events by 15% (95% CI 5.6-23.7%) relative to the control intervention.(16) Ensuring safe environment and safe practices applied by nurses reduced violent behavior of psychiatric patients.(17) In another study ward structure was associated with conflict rates,(18) the ward atmosphere and the relationships between patients and staff contribute to the improvement in symptoms and psychiatric patient functioning and satisfaction.(19) 

One-fourth of schizophrenia patients in acute wards showed self-harm and suicidal behaviors.(20) In the present study participants who underwent milieu therapy showed significantly decreased self-harm behavior compared to patients who underwent only routine therapy. In the present study milieu therapy ensured safe and secure environment by removing sharp objects from the patient environment, encouraged patient to express his negative emotions, taught safe and unsafe behavior, reinforced adaptive behavior. Bowers,(5) proposed a safe ward model to reduce conflict and containment rates in psychiatric wards. In this model the strategies included were special observation, patient autonomy and effective communication.

In the current study there was statistically significant reduction in general rule breaking behaviour of experimental group participants compared to control group participants. Experimental group participants’ wards had general rules displayed, taught time to time and sensitized with the consequences of breaking these rules. A study reviewed that there is relationship between ward rules and patient aggression.(21) 21 In the present study patient care givers acquired comprehensive knowledge about schizophrenia disorder, how to communicate with schizophrenia patients and how to manage their behaviors. Therapy provided a context for the care givers to realize that conflicting behaviors are merely symptoms of schizophrenic patients and it can be managed by effective communication, structuring daily activities and by modification of environment. The activities present in the intervention module not only help the participants to deal with the problems at hand but also prevent further potential problems and enhance the independence of the patients in their daily living activities. This study has provided preliminary evidence in the Indian context that milieu therapy was effective in reducing conflicting behaviors among schizophrenia patients. 

Though the study outcomes are encouraging, there are a few limitations. It is difficult to generalize the findings, as sample size was small. There was lack of long term follow up due to time constraints. The assessment and intervention was conducted for a limited time period during the day (8hours), while inclusion of the whole day (24 hours) will further strengthen the study results. The study used convenient sampling technique to prevent intervention contamination, though use of random sampling technique would lend further credentials to the study results.

This study concluded that schizophrenia patients who underwent milieu therapy intervention implemented by a nurse had statistically significant reduction in conflict rates relative to control group patients. Based on the findings of this study, Milieu Therapy is an effective component that should be considered as an integral part of any acute ward of psychiatric care setting for the schizophrenia patients.
